# Circadian Rhythmicity by Autocatalysis

**DOI:** 10.1371/journal.pcbi.0020096

**Published:** 2006-07-28

**Authors:** Arun Mehra, Christian I Hong, Mi Shi, Jennifer J Loros, Jay C Dunlap, Peter Ruoff

**Affiliations:** 1 Department of Genetics, Dartmouth Medical School, Hanover, New Hampshire, United States of America; 2 Department of Mathematics and Natural Science, University of Stavanger, Stavanger, Norway; 3 Department of Biochemistry, Dartmouth Medical School, Hanover, New Hampshire, United States of America; Lawrence Berkeley National Laboratory, United States of America

## Abstract

The temperature compensated in vitro oscillation of cyanobacterial KaiC phosphorylation, the first example of a thermodynamically closed system showing circadian rhythmicity, only involves the three Kai proteins (KaiA, KaiB, and KaiC) and ATP. In this paper, we describe a model in which the KaiA- and KaiB-assisted autocatalytic phosphorylation and dephosphorylation of KaiC are the source for circadian rhythmicity. This model, based upon autocatalysis instead of transcription-translation negative feedback, shows temperature-compensated circadian limit-cycle oscillations with KaiC phosphorylation profiles and has period lengths and rate constant values that are consistent with experimental observations.

## Synopsis

Circadian rhythms are a central feature of biological systems. In cyanobacteria, the clock involves three major proteins: KaiA, KaiB, and KaiC, with KaiC showing autophosphorylation in the presence of ATP. Remarkably, by incubating the purified Kai proteins with ATP, the clock can be reconstituted in vitro. The authors were intrigued by the simplicity of this oscillator and its connection to chemical oscillatory reactions, and saw the possibility for a realistic reaction kinetic representation. Their study represents a synthetic, predictive, and dynamic explanation for the in vitro KaiC circadian clock based on the self-amplifying response (“autocatalysis”) of autophosphorylating kinases. The presented model is based on existing biological and biochemical observations and recapitulates observed experimental features, including temperature compensation, which has been notably recalcitrant to explanation. The model provides several predictions including that period length and the ratio between phosphorylated and unphosphorylated KaiC in clock mutants are closely linked and related to the stability of the ternary complex formed between the three Kai proteins. The model also predicts the occurrence of bistability and that evolution can move simple bistable systems into oscillatory systems by changing only one rate constant.

## Introduction

Circadian rhythms are environmentally adaptive and homeostatically regulated oscillators found in many organisms. In cyanobacteria, fungi, flies, and mammals, transcriptional-translational negative feedback loops of clock genes have been identified [1,2] and modeled [3–6] as constituents of the circadian pacemakers. The simplest model organisms in which clocks are studied are the cyanobacteria [2]. As is the case in other species, these prokaryotes are known to show circadian rhythmicity due to negative feedback regulation of clock genes [7,8]. Together, KaiA, KaiB, and KaiC proteins broadly regulate the cyanobacterial transcriptome [9,10], and in vivo, these proteins appear to participate in a bona fide transcriptional-translational negative feedback oscillator [2].

Recent evidence, however, suggests that the Synechococcus elongatus PCC 7942 clock does not absolutely require such a negative feedback oscillator to generate phosphorylation rhythms in a key regulator, KaiC. Temperature-compensated oscillation in KaiC phosphorylation can occur in vivo without *kaiBC* mRNA accumulation, or in the presence of transcription and translation inhibitors [11]. Astoundingly, this self-sustainable and temperature-compensated oscillator can be reconstituted in vitro by incubating purified KaiC, KaiA, KaiB, and ATP [12]. We were intrigued by the elegant minimalism of this oscillator and its clear connection to chemical oscillatory reactions [4,5,13,14], and saw the possibility for a realistic mathematical representation. We therefore built a deterministic, basic model of this three-protein clock.

## Results/Discussion

Oscillation in KaiC phosphorylation is the best-observed parameter in this system and represents a key state variable for the clock in vivo. Thus we have sought to closely mimic this output in our study. KaiC is an enzyme with autokinase and autophosphatase activities [15,16]. KaiA enhances KaiC function [17] while KaiB diminishes the effect of KaiA on KaiC [16,18]. Nakajima et al. [12] suggest, given the dual function of KaiC and “cooperation between KaiA and KaiB,” that autonomous oscillation of KaiC phosphorylation might be achieved.

**Figure 1:**
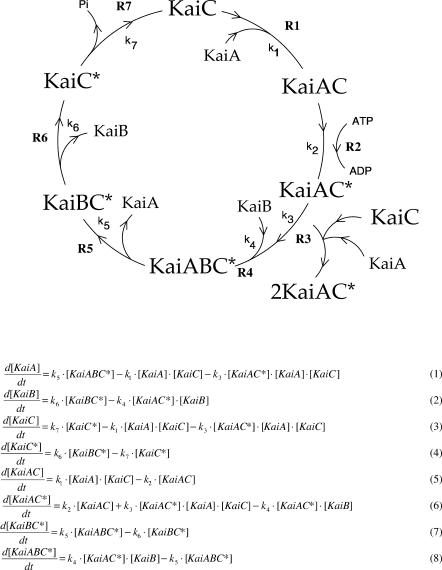
A Dynamical Model of In Vitro Rhythmic KaiC Phosphorylation See text for description.

We decided to explore this suggestion explicitly by establishing a basic model based on known biological and biochemical observations that did not involve transcription or translation. In Figure 1, we summarize key steps that we reasoned underlie the KaiC oscillator when ATP is provided in excess. It is well established that the three Kai proteins interact in all possible combinations [19] and, when possible, we have incorporated phase-specific interactions obtained from the literature [18]. The model (Figure 1), represented as a cyclic reaction diagram, contains seven processes (R1–R7 with rate constants k_1_–k_7_) depicting the proposed protein–protein interactions and phosphorylation–dephosphorylation events between the Kai proteins. KaiXY denotes the interaction between KaiX and KaiY proteins. In KaiC*, the asterisk indicates fully phosphorylated KaiC. KaiC without an asterisk represents largely unphosphorylated as well as low levels of incompletely phosphorylated KaiC molecules (for example, constituents of hypophosphorylated hexamers) [20]. In process R1, KaiC binds KaiA, forming KaiAC [21]. Since we have not explicitly formulated the dual phosphorylation events on KaiC, the model does not exclude the possibility that low levels of phosphorylated KaiC are required before KaiA efficiently binds KaiC as observed by Nishiwaki et al. [20]. Since KaiA facilitates the autokinase activity of KaiC [17], KaiAC rapidly converts to the fully phosphorylated form, KaiAC*, by process R2. Further, this step is consistent with the observation that KaiA inhibits KaiC dephosphorylation [16]. In the next step, R3, we propose that KaiAC* adopts such a conformation so that it can facilitate assembly and phosphorylation of free KaiA and KaiC into KaiAC*, extending the proposal by Kitayama et al. [18] that “KaiA enhances the accumulation of KaiC...by enhancing its autokinase activity in a processive manner” and that “phosphorylated KaiC may accelerate its binding to KaiA.” Interestingly, a possible “double-doughnut” conformation [22] of KaiC hexamers might be the basis for a scaffold-facilitated replication mechanism. In process R4, KaiB associates with KaiAC* to form the ternary complex KaiABC*. This is consistent with experimental data that demonstrate that KaiB interacts with KaiC only after KaiC has bound to KaiA [18,23]. KaiA's crystal structure [24] suggests that KaiB and KaiC binding may be cooperatively regulated, and this is consistent with our proposed stepwise binding. In process R5, KaiA is displaced from KaiABC*, and this reaction follows a proposal that KaiB might displace KaiA on KaiC due to a common binding site on KaiC [25]. The in vitro KaiB attenuation of KaiA stimulation of KaiC might be achieved by such a displacement [26]. When KaiA is no longer present in KaiABC*, we propose that KaiB dissociates from KaiBC* (process R6). Finally, in process R7, KaiC* utilizes its autophosphatase activity [15] and converts into KaiC. Although processes R6 and R7 might happen in either order or simultaneously, we preferred a sequential reaction order, because KaiB is largely in complex with phosphorylated KaiC [20]. Equations 1–8 (Figure 1) are the rate equations of the model.

**Figure 2:**
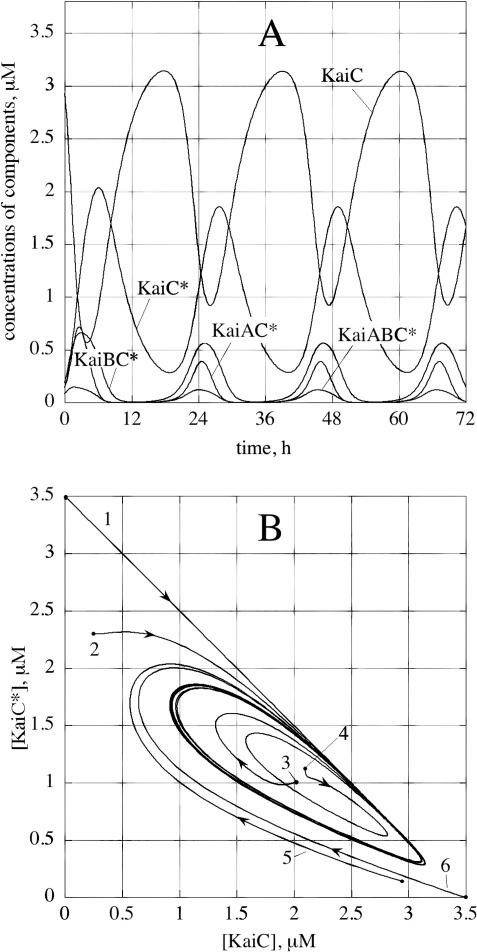
Limit Cycle Oscillations The rate constant values are: k_1_ = 1 × 10^−4^ μM^−1^ h^−1^, k_2_ = 0.4 h^−1^, k_3_ = 0.45 μM^−2^ h^−1^, k_4_ = 3.65 μM^−1^ h^−1^, k_5_ = 4.0 h^−1^, k_6_ = 0.9 h^−1^, and k_7_ = 0.18 h^−1^. (A) Unphosphorylated KaiC and phosphorylated KaiC (KaiC*) oscillate with the largest amplitudes. Oscillations of free unbound KaiA and KaiB concentrations are not shown, but they have amplitudes similar to the KaiBC* oscillations and peak at approximately the same phase as KaiC. (B) Six different approaches to the limit cycle, each obeying mass balances in total KaiA, total KaiB, and total KaiC, i.e., [KaiA]_tot_ = [KaiA] + [KaiAC] + [KaiAC*] + [KaiABC*] = 3 μM, [KaiB]_tot_ = [KaiB] + [KaiABC*] + [KaiBC*] = 1 μM, and [KaiC]_tot_ = [KaiC] + [KaiAC] + [KaiAC*] + [KaiABC*] + [KaiBC*] + [KaiC*]** =** 3.5 μM. (1) [KaiA] = 3 μM, [KaiB] = 1 μM, and [KaiC*] = 3.5 μM; (2) [KaiA] = 2.49 μM, [KaiABC*] = 0.137 μM, [KaiAC] = 0.0028 μM, [KaiAC*] = 0.369 μM, [KaiB] = 0.419 μM, [KaiBC*] = 0.444 μM, [KaiC] = 0.25 μM, and [KaiC*] = 2.3 μM; (3) [KaiA] = 2.54 μM, [KaiABC*] = 0.137 μM, [KaiAC] = 0.050 μM, [KaiAC*] = 0.269 μM, [KaiB] = 0.819 μM, [KaiBC*] = 0.444 μM, [KaiC] = 2.0 μM, and [KaiC*] = 1.0 μM; (4) [KaiA] = 2.74 μM, [KaiABC*] = 0.137 μM, [KaiAC] = 0.050 μM, [KaiAC*] = 0.069 μM, [KaiB] = 0.819 μM, [KaiBC*] = 0.444 μM, [KaiC] = 2.1 μM, and [KaiC*] = 1.1 μM; (5) [KaiA] = 2.7641 μM, [KaiABC*] = 0.0833 μM, [KaiAC] = 0.0036 μM, [KaiAC*] = 0.1490 μM, [KaiB] = 0.7280 μM, [KaiBC*] = 0.1887 μM, [KaiC] = 2.9296 μM, and [KaiC*] = 0.14557 μM; and (6) [KaiA] = 3 μM, [KaiB] = 1 μM, and [KaiC] = 3.5 μM.

The source of the oscillations is the autocatalytic reaction R3 of the phosphorylated KaiC–KaiA complex (KaiAC*, Figure 1) coupled with subsequent removal of the autocatalytic species and regeneration of dephosphorylated KaiC (reaction R7). The suggested autocatalytic formation of KaiAC* is typical for the kinetics of protein kinases showing autophosphorylation [27]. The autocatalysis permits sustained oscillations around a nonequilibrium unstable steady state as long as enough ATP is present to drive the autocatalytic phosphorylation (see Figure 2) which keeps the system far from thermodynamic equilibrium [28]. In the model, this is represented by treating the component processes as irreversible. However, in the absence of the autocatalytic step, no sustained oscillations appear possible, but damped oscillations may be observed in the approach to a nonoscillatory steady state [29].

The in vitro KaiC oscillator is based on the KaiA/KaiB-assisted phosphorylation/dephosphorylation of KaiC at *constant* protein levels. Therefore, unlike circadian oscillators based on transcription and translation [1], degradation reactions do not play any significant role in the in vitro KaiC oscillator.

Although the autocatalytic step in Figure 1 is represented as the termolecular process R3, we emphasize that this step can be readily broken up into a set of consecutive bimolecular reactions (see below) and by including explicitly multimeric forms of KaiA, KaiB, and KaiC. Although these additions lead to an even better quantitative description of the experimental data, the dynamic features of the autocatalytic loop are well represented by the model in Figure 1 on which we focus here.

Most of the model's rate constant values are not known apart from first-order KaiC phosphorylation and dephosphorylation constants (k_2_ and k_7_), which have been estimated [11,12] to be in the range 10^−3^ s^−1^ to 10^−4^ s^−1^ (3.6 h^−1^ to 0.36 h^−1^).

**Figure 3:**
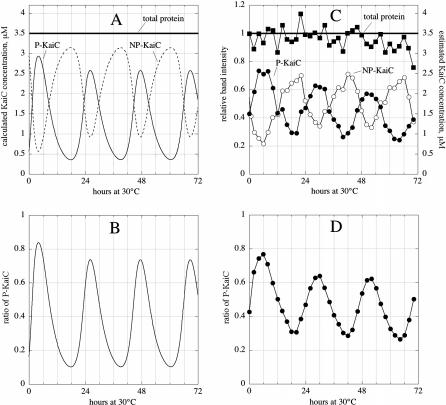
Comparison of Model Oscillations with Experimental Results Same rate constant values as in Figure 2. Initial concentrations as in curve 5, Figure 2B. (A) Calculated oscillations of total phosphorylated KaiC (P-KaiC = [KaiAC*] + [KaiBC*] + [KaiC*]), total unphosphorylated KaiC (NP-KaiC = [KaiC] + [KaiAC]), and their antiphase behavior. Total protein is P-KaiC + NP-KaiC. (B) Calculated oscillatory ratio of P-KaiC = P-KaiC/(P-KaiC + NP-KaiC). (C) Experimental oscillations of P-KaiC and NP-KaiC with estimated KaiC concentrations. Data replotted from [12]. (D) Experimental oscillatory ratio of P-KaiC = P-KaiC/(P-KaiC + NP-KaiC). Data replotted from [12].

**Figure 4:**
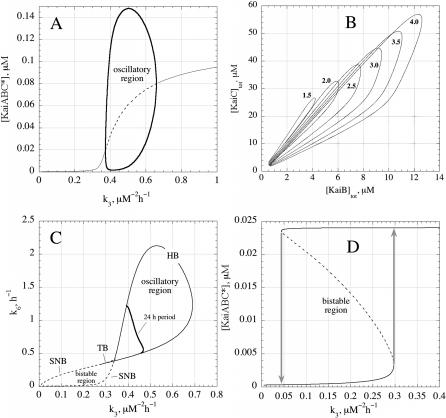
Oscillatory and Bistable Regions (A) Stable oscillations exist only for certain rate constant values as shown here for variations in k_3_ (0.373 μM^−2^ h^−1^ ≤ k_3_ ≤ 0.660 μM^−2^ h^−1^; all other rate constants as in Figure 2). The dashed line within the oscillatory region indicates the unstable steady state, which becomes stable outside the oscillatory region (solid lines). (B) The oscillations are crucially dependent on the total concentrations of KaiA, KaiB, and KaiC. The figure shows the oscillatory regions as closed loops in the concentration space of total KaiC ([KaiC]_tot_) and total KaiB ([KaiB]_tot_) when total concentrations of KaiA ([KaiA]_tot_ are varied from 4.0 μM to 1.5 μM (see numbers in graph). The model predicts that the region of oscillations shrinks as the total KaiA concentration is lowered. (C) The oscillatory and bistable regions (Figure 3A and 3B) are found within a so-called “cross-shaped diagram” shown here in the k_6_–k_3_ parameter space. Such diagrams have been observed in chemical oscillatory systems [39]. In more technical terms, HB denotes a Hopf bifurcation, SNB a Saddle Node bifurcation, and TB the Takens-Bogdanov bifurcation. The solid heavy line inside the oscillatory region indicates oscillations with a period length of 24 h. (D) The model can show bistability/hysteresis when for example k_6_ is reduced from 0.9 h^−1^ to 0.1 h^−1^. Steady state levels of KaiABC* are shown as a function of k_3_. The dashed line (0.0446 μM^−2^ h^−1^ ≤ k_3_ ≤ 0.297 μM^−2^ h^−1^) indicates unstable steady states and the bistable region. Solid lines show stable steady states.

A direct comparison of the model with experiments [12] is shown in Figure 3, in which rate constants have been used to get oscillations close to the observed experimental amplitude and to the experimentally observed phosphorylation and dephosphorylation constants [11,12]. One of the features we observe is that for a given set of rate constants oscillations occur in a relatively limited range of initial concentrations, illustrated by the bifurcation analysis shown in Figure 4A and 4B. The model also predicts the occurrence of bistability, for example when the rate constant of process R6 is reduced (Figure 4C). In such a bistable state, small perturbations may drive the system from one stable steady state to another and vice versa, crossing two different thresholds (Figure 4D). Based on this figure, it appears that subtle changes in only one or two steps may convert a bistable system into a self-sustained oscillator, and we speculate that such a conversion may have occurred during the early evolution of one or more circadian clocks.

Temperature compensation is an essential property of circadian rhythms. In 1957, Hastings and Sweeney [30] suggested that temperature-compensated oscillators contain reactions that have opposing effects on the period *P*. A kinetic analysis of temperature compensation showed [31] that the Hastings and Sweeney principle of opposing reactions can be formulated in terms of metabolic control theory [32], i.e., temperature compensation occurs *locally* (within a certain temperature interval), whenever the activation energy *(E_i_)* weighted sum of the control coefficients *C_i_* = ∂ln*P*/∂*lnk_i_* becomes zero, and the following *balancing equation* can be written:


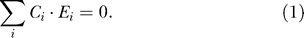


**Figure 5:**
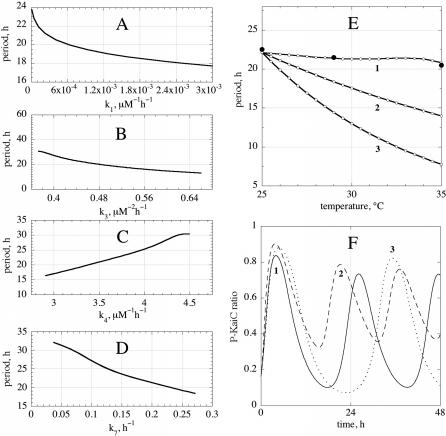
Parameter Sensitivities and Temperature Compensation (A–D) Period sensitivity analyses by varying the following selected rate constants (A) k_1_, (B) k_3_, (C) k_4_, and (D) k_7_. As k_i_ is varied, the other rate constants are kept constant at their initial values as described in Figure 2. (E) Temperature dependence of the period. Open circles show calculations, and solid circles show experimental results replotted from [1]. Each rate constant obeys the Arrhenius equation, k_i_ = A_i_ exp (−E_i_/RT), where E_i_ is the activation energy, R is the gas constant, and T is the temperature in Kelvin. A_i_ is the pre-exponential factor, which is also treated as a constant. Initial values of rate constants (Figure 2) are defined at 25 °C, and the period lengths are calculated for temperatures between 25 °C and 35 °C. (1) Temperature compensation (Q_10_ = 1.06) using the following activation energies: E_1_ = 40 kJ/mol, E_2_ = 35 kJ/mol, E_3_ = 28 kJ/mol, E_4_ = 43 kJ/mol, E_5_ = 40 kJ/mol, E_6_ = 25 kJ/mol, and E_7_ = 25 kJ/mol. (2) All activation energies have the average value (34 kJ/mol) from the calculation in (1) showing a Q_10_ of 1.58. (3) All activation energies are 80 kJ/mol, and the oscillator shows a Q_10_ of 2.85. (F) Dependence of period and P-KaiC ratio as a function of KaiABC* stability mimicking KaiC mutant behaviors. (1) Rate constants are as in Figure 2 (“wild-type behavior”); (2) Decreased P-KaiC ratios amplitudes and shorter periods (“short period KaiC mutant behavior”) are observed for a more stable KaiABC* complex relative to (1): k_4_ = 2.3 μM^−1^ h^−1^, k_5_ = 4.5 h^−1^, k_6_ = 1.0 h^−1;^ and (3) Long-period KaiC mutant behavior is observed when the KaiABC* complex is more stable compared to (1): k_4_ = 4.0 μM^−1^ h^−1^, k_5_ = 1.5 h^−1^, and k_6_ = 0.8 h^−1^.

Because Σ*_i_ C_i_* = −1 [32], and activation energies are positive, temperature compensation requires that one or several of the control coefficients need to be positive. Sensitivity analysis shows that for the rate constants given in Figure 2, the *C_i_*'s have negative values, but only process R4 has a positive control coefficient over the entire parameter space for which oscillations are observed (Figure 5A–5D), thus enabling temperature compensation (Figure 5E). Because the *C_i_*'s are not true constants (they depend on the other rate constants and temperature), Equation 1 is only approximately valid within a certain temperature interval, which is of physiological importance for the organism. Although many activation energy combinations are possible to satisfy Equation 1, the natural selection of a temperature-compensated set apparently occurred during evolution. Using the rate constant values for the oscillator shown in Figure 2, the model not only can show amplitude values that are close to experimental observations, but it also shows a close agreement between calculated and experimentally observed temperature behavior of the period.

Several KaiC mutant strains have period lengths ranging from 17 h to 28 h [12]. A possible explanation for the different period lengths and changed amplitudes in the phosphorylated KaiC (P-KaiC) ratio (amount of P-KaiC in relation to total KaiC) in these mutants appears to be related to the stability of fully phosphorylated KaiABC (KaiABC*), the only ternary complex formed in the system (process R4). For long-period mutants, the ternary complex is predicted to be formed more rapidly and/or be more stable (compared to wild type), which leads to oscillations with larger amplitudes in the P-KaiC ratio. This is in contrast with simulated short-period mutants, in which KaiABC* is not as easily formed and/or is less stable than in wild type (Figure 5F).

**Figure 6:**
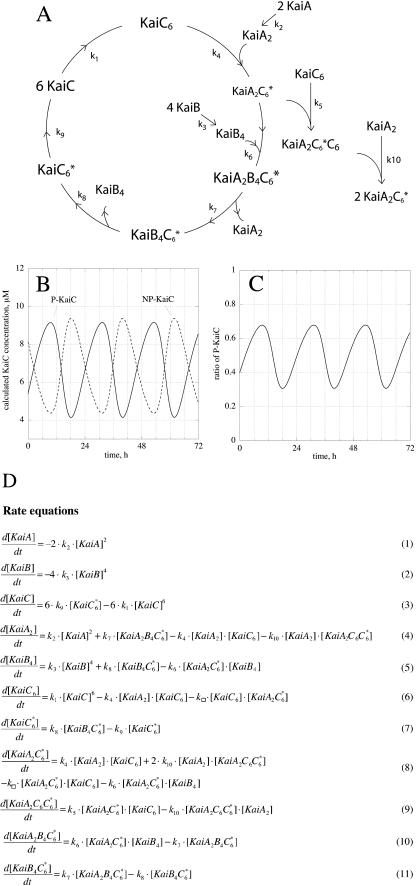
An Expanded Model of the KaiC Oscillator Containing Explicit Stoichiometries (A) KaiX*_k_*Y*_l_*Z*_m_* denotes a complex between *k* KaiX, *l* KaiY, and *m* KaiZ molecules. (B) Calculated oscillations of total phosphorylated KaiC (P-KaiC) and total unphosphorylated KaiC (NP-KaiC). (C) Calculated oscillatory ratio of P-KaiC = P-KaiC/(P-KaiC + NP-KaiC). (D) Rate equations of the model. The following rate constants and initial concentrations were used in (B) and (C): k_1_ = 0.1 μM^−5^ h^−1^, k_2_ = 0.1 μM^−1^ h^−1^, k_3_ = 0.1 μM^−3^ h^−1^, k_4_ = 0.001 μM^−1^ h^−1^, k_5_ = 1.8 μM^−1^ h^−1^, k_6_ = 2.5 μM^−1^ h^−1^, k_7_ = 1.0 h^−1^, k_8_ = 1.0 h^−1^, k_9_ = 0.1 h^−1^, and k_10_ = 40 μM^−1^ h^−1^. [KaiA] = 0.0161 μM, [KaiA_2_ B_4_C*_6_] = 0.0611 μM, [KaiA_2_C*_6_] = 0.0205 μM, [KaiA_2_C*_6_C_6_] = 7.4 × 10^−4^ μM, [KaiA_2_] = 0.9094 μM, [KaiB] = 0.1402 μM, [KaiB_4_C*_6_] = 0.0894 μM, [KaiB_4_] = 0.7395 μM, [KaiC] = 1.0288 μM, [KaiC*_6_] = 1.1805 μM, and [KaiC_6_] = 0.7256 μM.

Because termolecular processes such as reaction R3 appear to be unlikely, we tested an expanded version of the model by replacing process R3 by two consecutive bimolecular reactions leading to the autocatalytic formation of KaiA_2_C_6_* (Figure 6). In addition, we included in the model the formation of KaiA dimers [21,33] and KaiB tetramers [34], as well as KaiC hexamers [21,35]. Despite its increased complexity, the expanded model shows oscillatory behaviors (Figure 6B and 6C) similar to the basic model.

Recently, Emberly and Wingreen suggested an hourglass model [36] for the KaiC oscillator based on a set of nonlinear rate equations, predicting an exchange between KaiC hexamers during the day and the formation of KaiC hexamer clusters during the night. In contrast to the autocatalytic model presented here, oscillations in the hourglass model occur over a broad range of parameters and are independent of initial conditions [36].

Our model makes at least three specific, testable predictions that are not apparent from the biochemistry alone. Although these predictions are implicit in this paper, here we summarize them explicitly: (1) Varying initial concentrations of the Kai proteins should lead to a spectrum of responses from loss of periodicity to change in the actual period length. This prediction, especially the latter part, is not obvious from the biochemistry. (2) We have simulated behavior of a mutant KaiC. We predict that this mutant KaiC is specifically altered in its ability to form a KaiABC* ternary complex, and this should cause a predictable change in the amplitude of oscillation. (3) Finally, we propose that the major period-contributing parameter for temperature compensation is reaction R4. Our model predicts that if we introduce an antagonist of KaiB binding to KaiAC* (e.g., overexpression of the KaiAC* binding domain of KaiB), we should see, along with a decrease in period length (due to the reduction in free KaiB concentration), temperature under- or over-compensation dependent on whether the activation energy for the assembly of the ternary complex (E_4_) is increased or decreased, respectively.

As more experimental data and rate constant values from the KaiC in vitro oscillator appear, more detailed models may be formulated and tested. However, the advantage of “minimal” models like the one presented here is that they are easily applied and extendable. A classic example is the Oregonator model of the Belousov-Zhabotinsky (BZ) reaction [13]. Although this chemical oscillator contains only three initial substrates in an acidic solution, the underlying chemistry is very complex. Yet, the use of the Oregonator model, which reduces the complex chemistry into a three-variable model, was able to predict and describe most of the system's astonishing behaviors including excitability, bistability, and chaos [13,14]. Although positive feedback loops are not common metabolic control mechanisms, our model suggests that the in vitro KaiC phosphorylation rhythm is a circadian oscillator that is driven by autocatalysis. In it, we see a new relationship between nonlinear chemical dynamics and chronobiology.

## Materials and Methods

The model's differential equations were solved numerically using the FORTRAN subroutine LSODE [37]. Stability and sensitivity analyses were done with the software tool XPPAUTO [38].

## Abbreviations

KaiABC*: fully phosphorylated KaiABC ternary complex
P-KaiC: phosphorylated KaiC
